# A Hierarchical Modeling Framework for Multiple Observer Transect Surveys

**DOI:** 10.1371/journal.pone.0042294

**Published:** 2012-08-08

**Authors:** Paul B. Conn, Jeffrey L. Laake, Devin S. Johnson

**Affiliations:** 1 National Marine Mammal Laboratory, Alaska Fisheries Science Center, National Marine Fisheries Service, Seattle, Washington, United States of America; University of California, Berkeley, United States of America

## Abstract

Ecologists often use multiple observer transect surveys to census animal populations. In addition to animal counts, these surveys produce sequences of detections and non-detections for each observer. When combined with additional data (i.e. covariates such as distance from the transect line), these sequences provide the additional information to estimate absolute abundance when detectability on the transect line is less than one. Although existing analysis approaches for such data have proven extremely useful, they have some limitations. For instance, it is difficult to extrapolate from observed areas to unobserved areas unless a rigorous sampling design is adhered to; it is also difficult to share information across spatial and temporal domains or to accommodate habitat-abundance relationships. In this paper, we introduce a hierarchical modeling framework for multiple observer line transects that removes these limitations. In particular, abundance intensities can be modeled as a function of habitat covariates, making it easier to extrapolate to unsampled areas. Our approach relies on a complete data representation of the state space, where unobserved animals and their covariates are modeled using a reversible jump Markov chain Monte Carlo algorithm. Observer detections are modeled via a bivariate normal distribution on the probit scale, with dependence induced by a distance-dependent correlation parameter. We illustrate performance of our approach with simulated data and on a known population of golf tees. In both cases, we show that our hierarchical modeling approach yields accurate inference about abundance and related parameters. In addition, we obtain accurate inference about population-level covariates (e.g. group size). We recommend that ecologists consider using hierarchical models when analyzing multiple-observer transect data, especially when it is difficult to rigorously follow pre-specified sampling designs. We provide a new R package, hierarchicalDS, to facilitate the building and fitting of these models.

## Introduction

Transect surveys are often used to sample animal populations and are a central component of many inventory and monitoring programs. In such surveys, an observer travels along a set of lines or visits a finite collection of points, recording all animals they encounter within a fixed distance of the line (or point). If all animals within this strip are encountered, researchers can make inferences about abundance over a larger area by employing standard design-based sampling protocols [1]. On the other hand, if some animals are missed (as is almost always the case), approaches are needed to correct for probabilities of detection (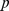) that are less than one.

Distance sampling is one potential avenue for correcting for incomplete detection of animals in fixed area polygons. In its canonical form (e.g. [2], [3]), distance sampling is a simple extension of quadrat sampling, whereby an observer notes the perpendicular distance of animals or groups of animals from the centerline (or radial distance from a point). Analysts then fit models to distance data that allow them to express the probability of detecting a group of animals as a function 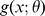 of distance 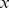, where 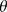 are parameters to be estimated. Total abundance in the surveyed area can then be estimated as


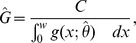


where 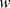 is half the transect width. In practice, 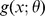 must often assumed to be 1.0 at distance 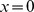 when transects are traversed by a single observer for parameters to be identifiable.

When animals are detectable from the air, from vessels at sea, or by other means (e.g. avian auditory counts), distance sampling provides a way to correct for imperfect detection in animal surveys without having to physically capture and mark animals. Correcting for imperfect detection is necessary when estimating absolute abundance, and is also viewed by many as an essential component of trend estimation because trends in detectability are typically confounded with trends in abundance unless detectability is explicitly accounted for [4], [5]. For these reasons, practitioners routinely use distance sampling in contemporary animal transect surveys.

Researchers have extended conventional distance sampling to account for a variety of complications that arise in real life sampling scenarios. Several studies have utilized multiple observers to relax the assumption of complete detectability on the transect line [6], and to model heterogeneity in detection as a function of distance [7]–[9]. These features are common in many datasets, and can cause negative bias in abundance estimates if unaccounted for. Other authors have extended conventional distance sampling to model the effects of individual and external covariates on detection probabilities [10]–[12]. As with conventional approaches to distance sampling, estimators are often obtained via a two-stage approach, whereby analysts first formulate models for multiple-observer and distance data, and then estimate abundance using a Horvitz-Thompson-like estimator [13], [14].

Several authors have recently proposed using hierarchical, Bayesian models in place of likelihood or moment-based estimators to analyze distance sampling data [15]–[20]. The appeal of using hierarchical models is undeniable, as they permit straightforward inference about the relationship of animal abundance to habitat-specific covariates [21], and allow parsimonious relationships to be specified among abundance parameters in time and space [22]. For instance, Moore and Barlow [18] and Chelgren et al. [19] recently used hierarchical models to account for strata and year effects on animal densities when analyzing line transect data.

**Figure 1:**
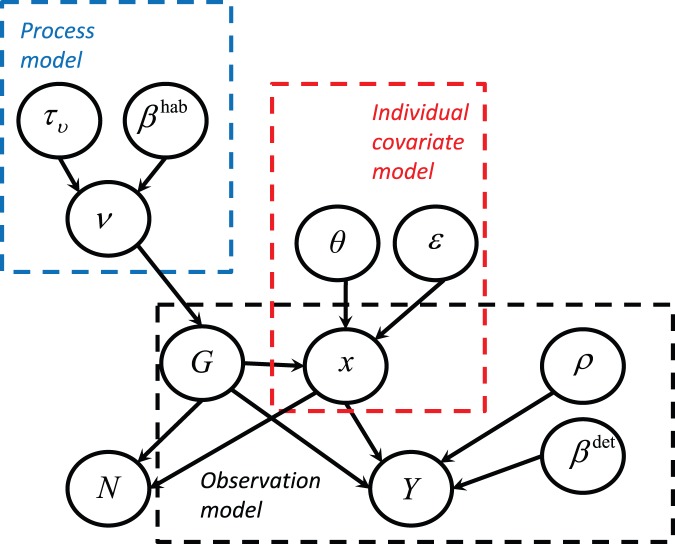
Directed, acyclic graph (DAG) of the (areal) hierarchical model for distance data. Individual nodes indicate a parameter or vector of parameters, and arrows represent conditional dependence. Notation is defined in Table 1.

Thus far, attempts to analyze line transect data with hierarchical models have focused on single observer data. In this paper, we develop hierarchical models for double observer data that permit habitat covariates to influence abundance intensity, while simultaneously modeling effects of covariates on detection probability. Since double observers are employed, these models allow for 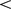
100% detection on the transect line (by contrast, previously developed hierarchical modeling approaches had to assume 100% detection on the line). Our approach can also accommodate increasing dependence among observer detections as a function of distance. Building upon previous work by Durban and Elston [23] in the context of mark-recapture modeling, our approach for modeling detections and abundance at the transect level is based on data augmentation [24], [25], using a reversible jump Markov chain Monte Carlo (RJMCMC) algorithm [26], [27] to sample abundance and individual covariates.

Our modeling approach is applicable to sampling programs for a variety of taxa; here, we focus on describing a generalized hierarchical modeling framework, developing user friendly software, and demonstrating the viability of our approach. After describing our proposed model, we use a small simulation study to verify that it provides reasonable inference about abundance for multiple species with different habitat preferences. Finally, we analyze data from a known population of golf tees that were sampled via a double observer distance sampling protocol. Golf tee clusters varied by the number of tees in each cluster, by color, and by level of exposure, allowing us to fit models that expressed detection probability as a function of covariates and to estimate posterior distributions for these covariates. In contrast to most population surveys, truth is known for this dataset and provides a verifiable test of our modeling framework.

**Table 1:** Parameter and data definitions.

Parameter	Definition
*N*	Total animal abundance in the study area
*G_j_*	Number of groups of animals located in area *j*
*v_j_*	The log of abundance intensity in area *j*
*τ_v_*	Precision of log of abundance intensity; used to impart overdispersion relative to the Poisson distribution
*λ_j_*	Abundance intensity in area *j* ( = exp(*v_j_*))
*β* ^hab^	Parameters of the linear predictor describing variation in the log of abundance intensity as a function of habitat covariates
*Z_ijk_*	The value of the *k*th individual covariate associated with group 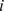 in transect *j* (for groups of animals never observed)
*θ*	Parameters describing the distribution of individual covariates at the population level
*β* ^det^	Parameters of the linear predictor describing variation in the probit of detection probability as a function of observer and individual covariates
*ρ*	Parameter describing increasing correlation between *Y_ij_*_1_ and *Y_ij_*_2_ as a function of distance when there are double observers
**Data**	**Definition**
*Y_ijk_*	Bernoulli response variable for whether the *i*th group in the *j*th transect was observed by observer *k*
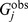	Number of groups observed by at least one observer during transect *j*
*O_j_*	Number of observers present when sampling transect *j*
*Z_ijk_*	The value of the *k*th individual covariate associated with group *i* in transect *j* if it was actually observed
*X* ^hab^	Design matrix associated with habitat model
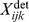	Design matrix associated with the detection model for the *i*th group in the *j*th transect and observer *k* (note dependence on *Z_ijk_*)
*S_j_*	Label for grid cell *j* (*J* of which are completely covered by transects, and *L*−*J* of which are unsampled cells).
*A_j_*	The area of grid cell *j* (perhaps scaled to its mean)

Parameters and data used in the hierarchical model for distance data.

## Methods

### Hierarchical Model

We propose a hierarchical model for distance sampling data consisting of several conceptually distinct components (Figure 1). Writing the model hierarchically, we can use conditioning to treat these components separately. Letting the notation 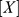 define the probability distribution or mass function of 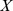, 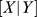 denote the conditional probability of 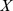 given 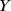, we (symbolically) write the likelihood of the hierarchical model as


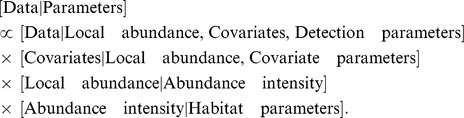


If conducting a Bayesian analysis, the posterior distribution is then proportional to


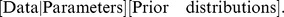


Given samples from the posterior, one can make posterior predictions of total abundance, so we might include another component 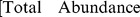 | 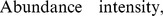 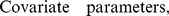 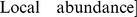 as well. Below, we treat each of these components in turn. Notation is largely defined in the text, but is also provided for convenience in Table 1.

**Figure 2:**
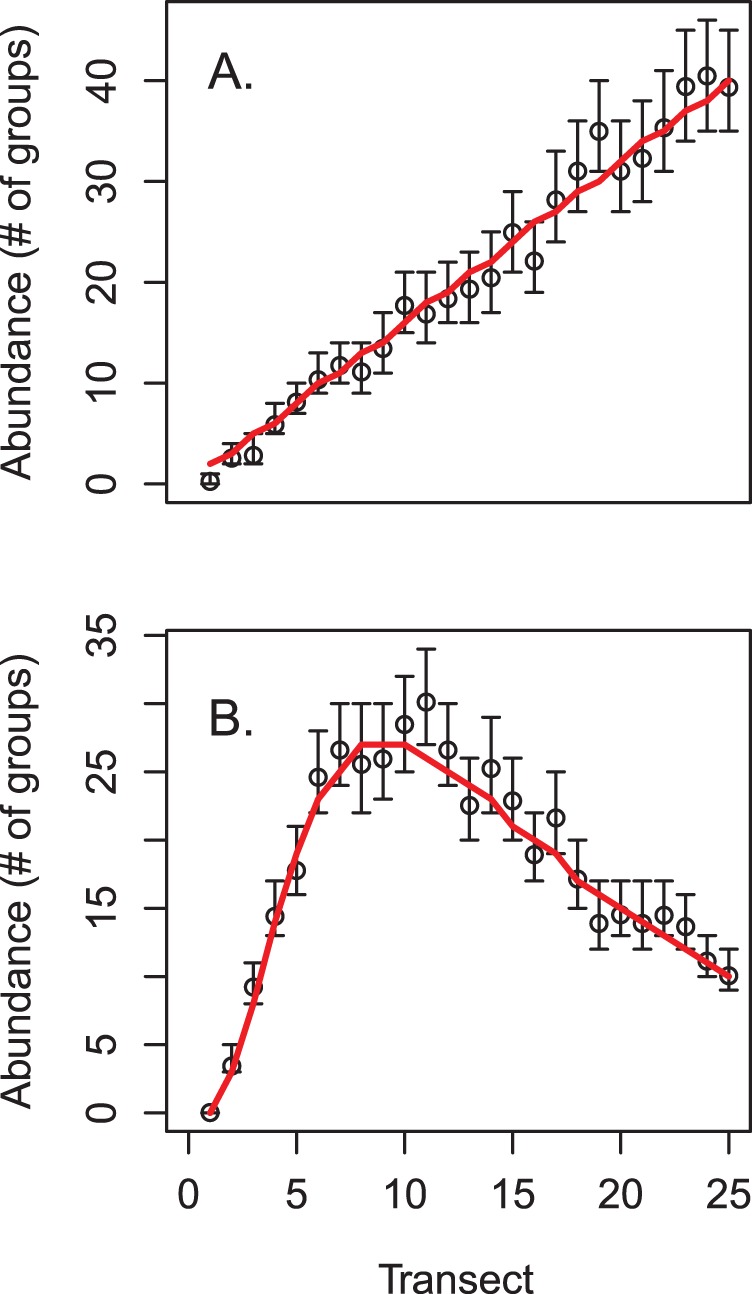
True and estimated population size for simulated data. True abundance is indicated in red, with posterior means and estimated 95% credible intervals for abundance indicated by circles and brackets, respectively. Panel (A) gives results for the simulation with linearly increasing abundance, while panel (B) gives results for the simulation with a quadratic relationship between abundance and a habitat covariate.

### Observation (Data) Model

The data collected in multiple observer transect surveys consist of a collection of binary observations, 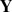, and covariates, 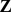. Detections (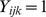) and non-detections (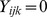) are recorded for each observer 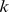 for the 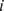
th group of animals encountered on transect 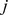. Covariates thought to influence detection probability may also be recorded, with 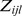 giving the value of covariate 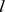 for the 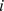
th group of animals encountered in transect 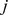. In practice, 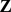 may include both transect specific covariates (e.g. survey conditions) or covariates associated with individual groups of animals (distance, group size, species, etc.).

**Figure 3:**
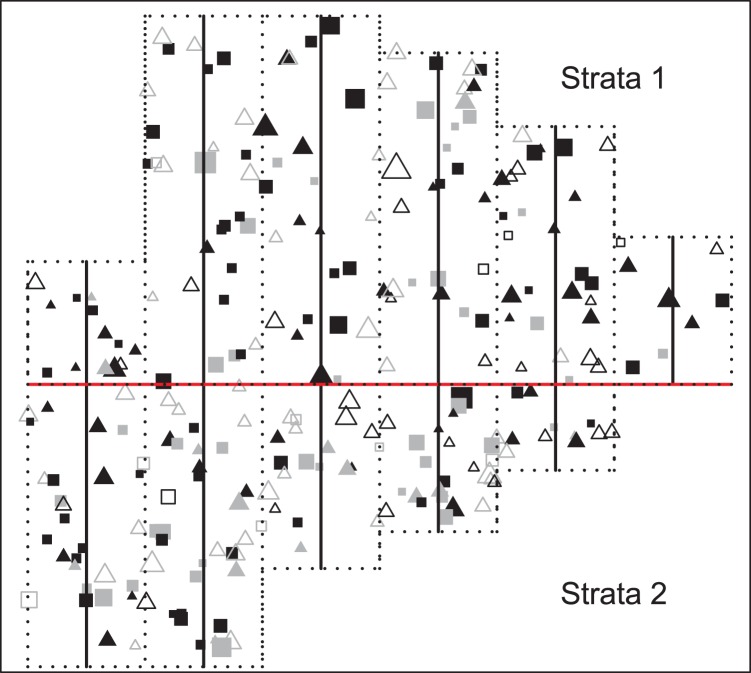
Representation of golf tee population. Each symbol represents a different group of golf tees, with dark symbols representing yellow tees and gray symbols representing green tees. Groups that were observed by at least one observer are indicated by solid symbols, while open symbols indicate groups that were never observed. Squares represent tee groups that were exposed above surrounding grass, while triangles represent unexposed groups. Group sizes are indicated by the proportional size of each symbol, with the smallest symbols representing groups of 1 animal, and the largest symbols representing a group of 8 individuals. Transect lines are represented by solid black lines, with dotted lines giving survey area boundaries and demarcating the areas surveyed by each transect. The red line serves as the strata boundary (points north comprise the northern stratum).

Suppose for the moment that we also knew the total number of groups present in the area associated with transect 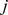, 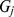, as well as covariate values for each group. In this case, we could augment the total number of observed groups, 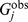 with an additional 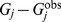 observations for which the 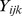 were all zero. We adopt this convention here, leaving it to a later section to describe the procedure by which 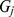 and the missing covariates are estimated.

**Figure 4:**
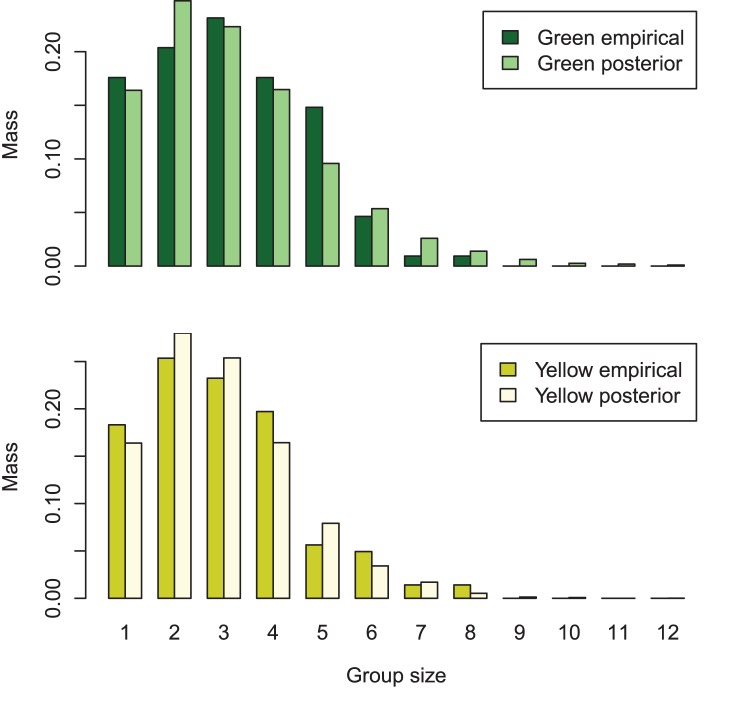
Empirical and posterior predictive distributions of golf tee group size. Bar plots representing the probability mass for group size in the golf tee experiment. Empirical distributions correspond to the actual distribution of group size used in the experiment, while posterior distributions represent estimated posterior predictive distributions obtained after analyzing data with our hierarchical model.

Conditional on 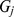 and 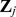, we assume that observations 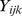 are Bernoulli distributed, with success probabilities 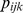. We model the 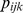 using a probit link function, expressing them as a function of covariates in a manner analogous to generalized linear models [28]. We also allow for increasing dependence among observers as a function of distance by allowing for correlation between 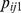 and 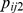 on the probit scale. Specifically, assuming two observers, we have


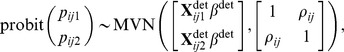


where 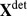 gives a design matrix incorporating any desired covariates for detection probability, and 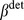 gives a vector of regression coefficients. Correlation (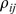) is set to be a function of distance by allowing 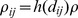, where 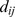 is the distance value associated with the 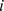
th group in the 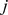
th transect and 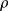 is an estimated parameter. For continuous data, we suppose that 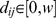, and for binned data, we suppose that distance bins are represented by finite integers with 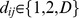 with 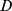 being the farthest distance bin. Correlation could potentially be a function of other covariates as well; however, there is typically limited information with which to estimate it. In the following development, we assume that correlation changes linearly on the probit scale as a function of distance from the observer. With binned distance data (i.e. when observers record distance as falling into one of a finite collection of distance intervals), we suppose that


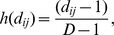


and with continuous distance data,


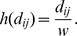


In both cases, 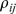 is assumed to be zero on the line (or in the first distance bin). This assumption, termed “point independence” by several authors [7]–[9], anchors the correlation function at distance zero and makes observer dependence parameters identifiable. This formulation also allows increasing dependence of observations as a function of distance, a common phenomenon in distance sampling [7]–[9].

**Figure 5:**
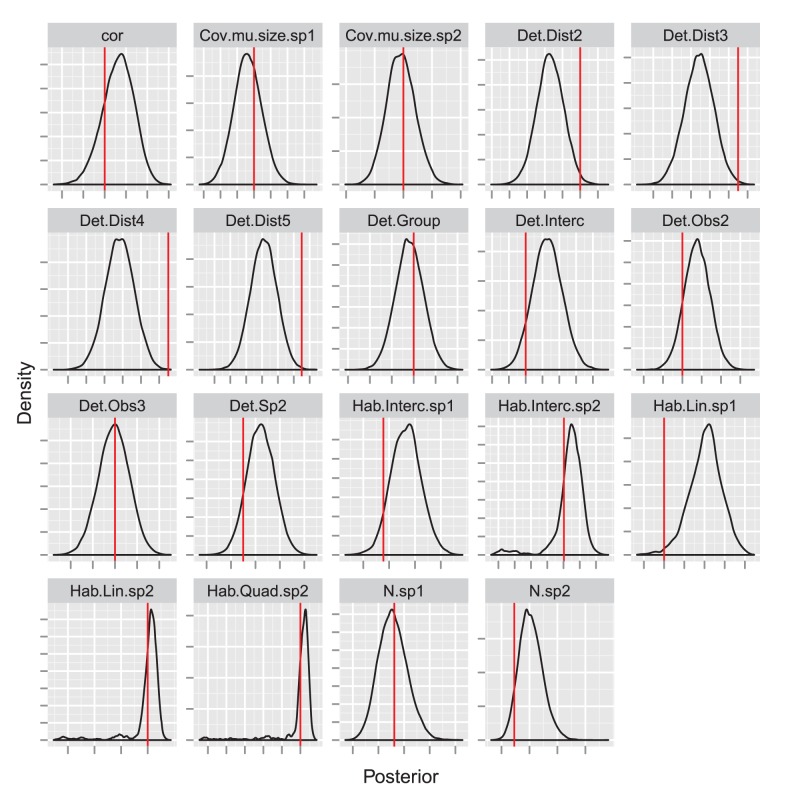
True values and estimated posterior distributions for simulated data. Kernel density estimates of marginal posterior distributions are indicated in black, with true values used to simulate data indicated by red, vertical lines. Parameters indexed by “Cov” give covariate parameters, “Det” give detection parameters, “Hab” give habitat parameters, and “N” gives abundance. The first panel (“cor”) gives an estimate of the observer dependence parameter. Species specific parameters are indexed by “sp1” (for species one) or “sp2” (species two).

**Figure 6:**
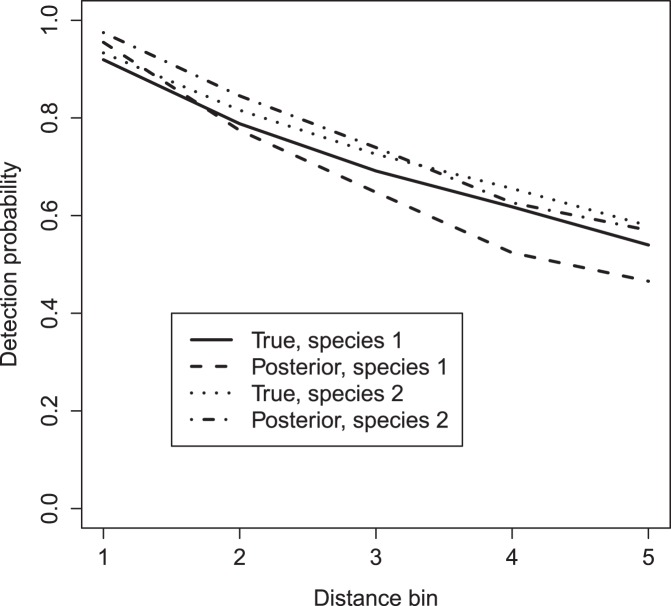
True and estimated detection functions for simulated data. Detection functions for each species are based on mean group sizes for each species (4 and 2, respectively), and are made for observer 2 (who had an intermediate detective ability).

Like the (more popular) logit link function, the probit link function provides a transformation from 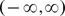 space (the space of the linear predictor) to 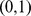 space (probability space). However, use of a probit link allowed us to employ the computationally efficient posterior sampling algorithm suggested by Albert and Chib [29]. In particular, we augment each detection with a latent variable, 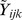, where 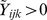 if and only if 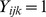. Then, we suppose that


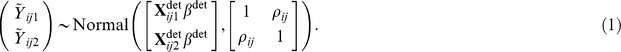


With multiple observers, conditioning on one value of 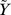 reduces the probability density from a bivariate to a univariate distribution. In particular,


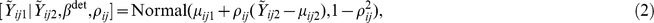


where 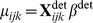. The full conditional distribution for 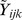 is then a truncated normal, which can be updated within MCMC using a Gibbs step; the 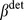 can also be updated directly with Gibbs steps (see Text S1 for further details).

### Covariate Model

Some covariates thought to influence detection probability are collected at the transect level (e.g. survey conditions), and are therefore known for all potential groups of animals (observed and unobserved). However, for covariates associated with individual groups of animals (e.g., distance, group size, species), an underlying model is needed to link the observed covariates (for observed groups) to unobserved covariates (for unobserved groups). We model these individual covariates as having arisen from a parametric distribution, possibly with overdispersion. In general, let the covariate 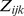 have the distribution


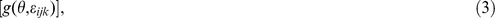


where 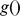 specifies an arbitrary function, 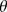 gives hyper-priors, and 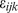 specifies a random effect. We have implemented a number of such distributions in our accompanying R package, hierarchicalDS. For instance, the user can choose 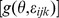 to be a uniform, multinomial, Poisson, zero-truncated Poisson, overdispersed Poisson, or overdispersed zero-truncated Poisson distribution. The overdispersed versions of the Poisson distribution are modeled as in [30]; namely


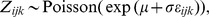


where 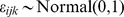 and 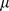 and 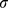 are parameters to be estimated. The zero-truncated versions of the Poisson distribution are important for modeling covariates such as group size, which are by definition 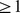 [31]. One can also choose to fix the parameters of these distributions, or to specify hyper-prior distributions. For further information, see accompanying R package.

### Process Model

Let 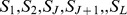 define a partition of the study area, where 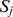, 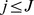, denote areas covered by individual transects, and 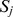, 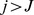, denote unsampled areas. In practice, these cells may be irregularly shaped, or with different effective areas covered, 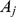. We suppose that the number of groups of animals located in the area encompassed by area 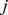, 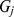, is Poisson distributed with intensity parameter 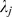. Overdispersion, if present, can be accommodated in the specification of 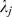. We model species-habitat relationships by writing abundance intensity as a function of habitat covariates (an intercept only model can be specified if no such covariates are collected). Letting 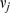 be the log of abundance intensity in 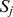, we impose the following model:


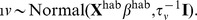


Here, 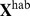 gives a design matrix incorporating any predictor variables for abundance, 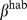 gives a vector of regression coefficients, and 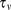 denotes precision of the process model. The resultant Poisson intensity is then 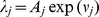. In practice, we find it convenient to scale 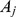 to its mean rather than use absolute values. This formulation incorporates habitat covariates in a manner analagous to generalized linear models [28], but also includes possible overdispersion relative to the Poisson distribution, a prevalent feature in ecological datasets. Such overdispersion can lead to left-skewed distributions for total abundance, similar to the lognormal distribution often assumed in conventional distance sampling [3].

### Posterior Predictions of Abundance

The model as written focuses on abundance of *groups*. In contrast, population managers often require estimates of density or abundance that reference the number of unique individuals inhabiting an area of interest. For surveyed cells (i.e., 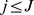), abundance can simply be calculated as 
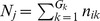
, where 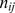 is the group size associated with observation 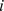 in the area covered by transect 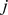 (which is itself a modeled covariate).

For areas that are unsurveyed (that is, 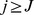), abundance 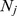 can be sampled using the parametric model selected for group size. For the zero-truncated Poisson model,


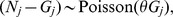


while for the zero-truncated overdispersed Poisson model,


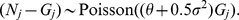


Posterior predictions of total abundance are then calculated as 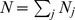.

### Prior Distributions

Bayesian analysis requires specification of prior distributions for 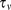, 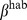, 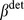, and 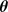. We chose a conjugate 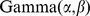 prior for 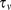, so that full conditional distributions were available in standard form and could be sampled directly with Gibbs sampling. In all subsequent analysis, we chose 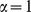 and 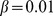 to put ample weight on plausible parameter values.

We gave the 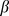, which are analogous to regression parameters, vague 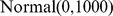 prior distributions, which is a common strategy in regression problems (e.g. [32], Chapter 14). By contrast, hyper-priors for individual covariates incorporated greater precision to improve sampling efficiency. In our two subsequent examples, we incorporated a 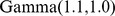 distribution for the log of group size, and a 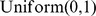 distribution for 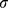 (the standard deviation for log group size random effects). For the simulation study and golf tee analysis, species and exposure were modeled with a Dirichlet(10,10) distribution, the conjugate prior for the categorical distribution. Pilot analyses suggested little sensitivity of results to our choice of prior distributions.

### Data Augmentation and Bayesian Inference

As suggested previously, the primary challenge in implementing a complete data model for multiple observer transect surveys was in jointly sampling local abundance and individual covariates. We chose to implement a reversible-jump algorithm (RJMCMC) to sample abundance at the transect level, in a manner similar to Durban and Elston [23]. The RJMCMC approach is commonly used in cases where the dimensionality of the parameter space is unknown (in our case, abundance is unknown) [27], and provides a mechanism to grow or shrink the number of parameters during posterior sampling. In our case, RJMCMC consists of several steps, including (1) additions and deletions of unobserved animals, (2) resampling of covariate values for unobserved animals, and (3) sampling of 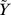 values for new additions. These steps were conducted independently for each transect since realizations for each transect are conditionally independent; thus without loss of generality, we present sampling details for a single transect.

Specification of the complete data model starts with specifying an integer 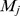 that serves as the upper limit for the number of groups of animals present in the sampled area of transect 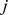. In practice, this integer can be increased if it is found that the posterior group abundance runs up against this bound [23], [25]. However, choosing too large of a value can dramatically increase computing time.

Addition and deletion steps consist of increasing or decreasing the value of 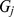, and are accomplished as follows:

- Propose a new value for 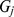, 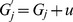, where 
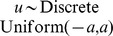
, and 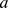 is a tuning parameter set to achieve a target acceptance rate of 0.3–0.4.
- Accept proposal with probability 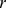, where

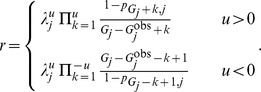


Here, 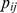 equals the probability that group 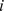 of transect 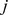 is observed by one or more observer. If there is only one observer on transect 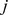, this probability is simply


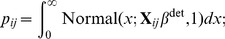


if 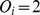,


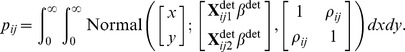


This formulation, including the integral and specification of variance as 1.0, corresponds to the inverse probit link function [33].

The Metropolis ratio, 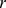, follows directly from a sampling model where the likelihood of observing 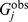 individuals out of 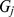 total animals is


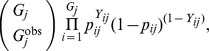


where 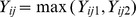. Link and Barker [16] used a similar model formulation in their complete data representation of distance data.

Following the addition/deletion step, the next step in our RJMCMC estimation scheme is to resample individual covariates. For each such covariate, there are two categories of values to update: (1) covariates for which a group of animals were in the population and never observed, and (2) latent groups not currently belonging to the population. Letting 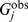 denote the number of groups observed by at least one observer in transect 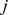, and 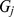 be the total number of groups for transect 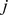 at the previous iteration of the Markov chain, the full conditional distribution for a given covariate 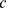, 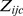 is given by


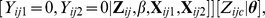


for 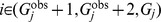, which is just the observation model (e.g. Eq. 1) multiplied by the prior distribution of the individual covariate. We use a Metropolis-Hastings step to sample from this distribution.

For “pseudo-groups” 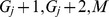 (i.e., groups of animals not currently in the population who might be added during a future RJMCMC step), we simulate covariates directly from Eq. 3. These distributions are used in place of the pseudo-prior distributions suggested by Durban and Elston [23]. The difference between our approaches is that in the present work we obtain posterior samples of the parameters specified in 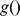 (e.g. the parameters describing the underlying covariate distribution), while Durban and Elston fix these parameters. Simulations (see below) suggest that this approach results in estimates with reasonable properties, and also bypasses the need to tediously tune pseudo-prior distributions. Assuming random placement of transect lines, we selected a uniform distribution a priori to simulate pseudo-group distances.

Estimation of remaining model parameters (conditional on a set level of abundance) proceeded by cyclical sampling of model parameters from their full conditional distributions [32]. In particular, we employed a combination of Gibbs and Metropolis-Hastings steps for posterior sampling. For Metropolis-Hastings steps, candidate parameter values were sampled from uniform distributions centered at the previous iterations parameter value and with a range chosen to achieve an acceptance rate of 30–40% as suggested by Gelman et al. [32]. For further details, see Text S1.

### Computing

We developed generalized computing code to conduct MCMC estimation, which we implemented in the R programming environment [34]. This code has been incorporated into the R package, HierarchicalDS. The package, which includes code, help files, and an example analysis, is currently available on the Comprehensive R Archive Network (CRAN; http://cran.r-project.org/).

**Figure 7:**
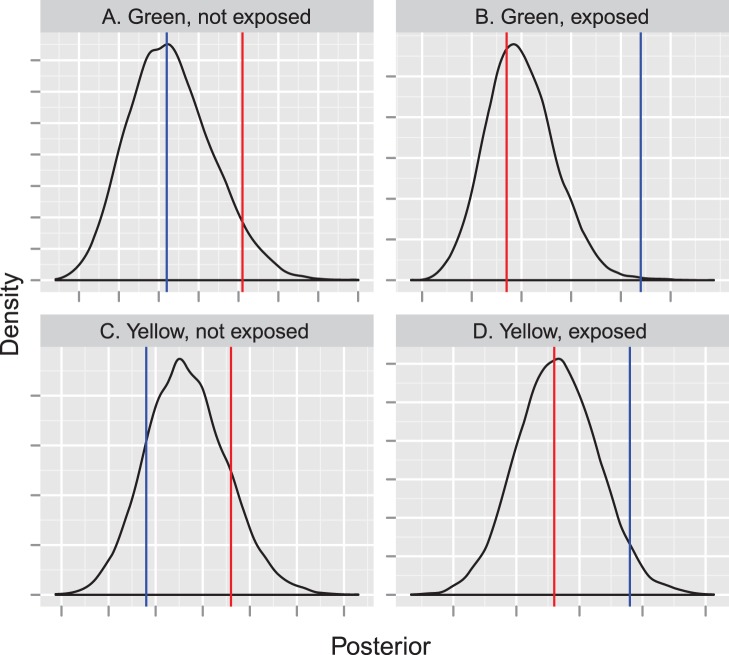
Posterior distributions for the abundance (number of groups) of golf tees of different types. Kernel density estimates of posterior distributions are in black, while true values are represented by red vertical lines, and estimates from a conventional mark-recapture distance sampling analysis (see Laake and Borchers [7]) are presented in blue.

### Examples

#### Simulated data

We first used simulation to verify that our modeling approach provided reasonable estimates of abundance and related parameters. In particular, we generated a double-observer distance sampling dataset for two species with different habitat preferences and covariate values, but with a common detection function. For the first species, expected abundance increased linearly with an arbitrary covariate (here, transect number); expected abundance of the second species had a quadratic relationship with the covariate (Fig. 2).

**Figure 8:**
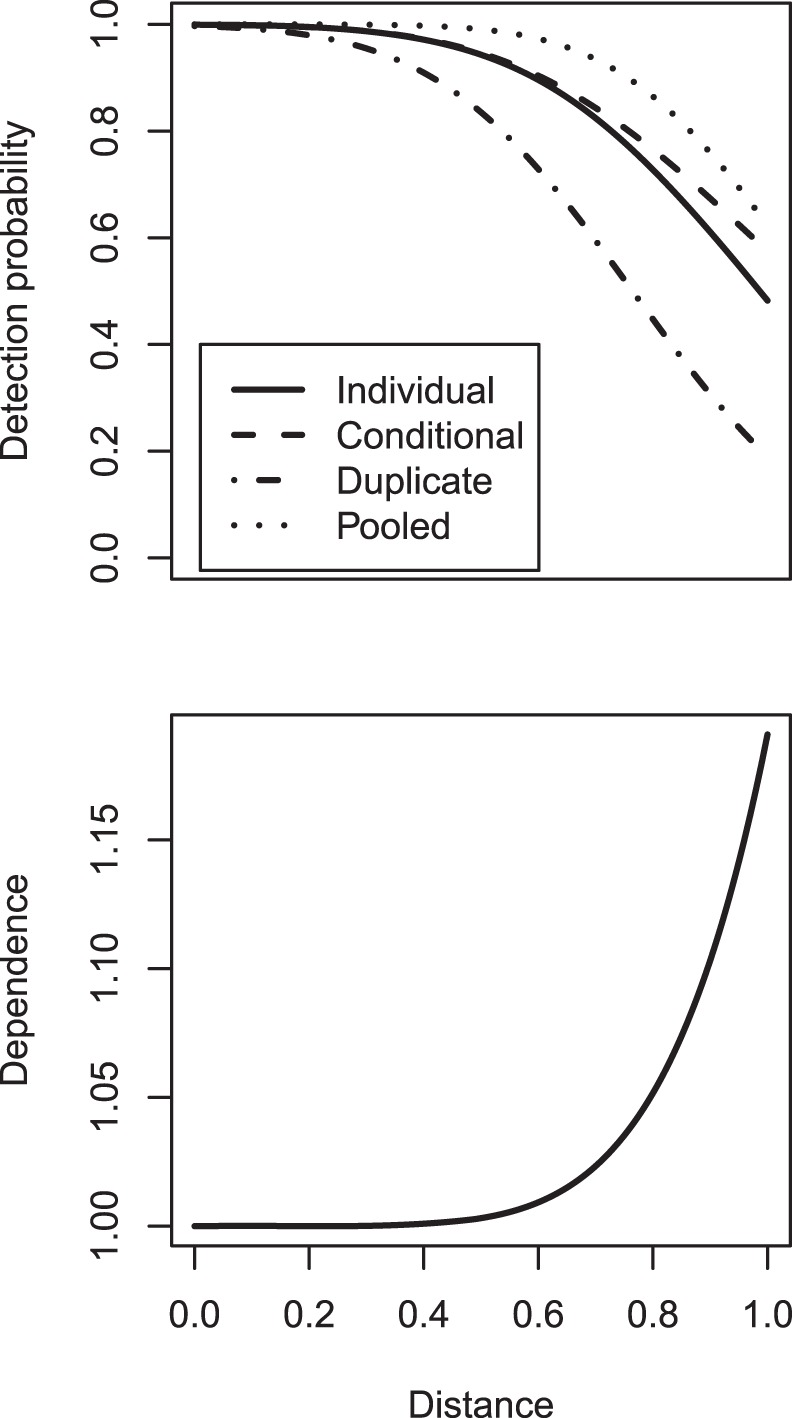
Implied detection probability and observer dependence for covariates maximizing dependence. The top panel gives detection probability curves for the set of covariates that maximize observer dependence (observer = 2, group size = 1, exposure = 0, species = “green”). “Individual” specifies detection probability for observer 2 only; “Conditional” gives the probability of detection for observer 2 given that the group was detected by observer 1; “Duplicate” gives the probability of detection by both observers; “Pooled” gives the probability of detection by at least one observer. The bottom panel represents dependence, as summarized by the parameter 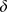 (see [9]) for the same set of covariates.

A total of 25 transects were simulated, each of which had two observers assigned. In all cases, observers were picked randomly from a pool of three total observers with different underlying detection probabilities. The assumed detection function was made to be a function of observer, distance, species, and group size. Correlation between observers was modeled as a linearly increasing function of distance on the probit scale, with a maximum value of 0.5 at the farthest observable distance.

**Table 2:** Modeling choices and justification.

Modeling construct	Choice	Alternatives	Advantages
*Point independence*	Bivariate normal distributionwith correlation as a functionof distance	Individual random effect	Not needed when there is one observer
*Detection model link function*	Probit	Logit, complimentary log-log	Simplifies Bayesian computation through Albert and Chib algorithm [29]
*Data representation*	Complete data likelihood/data augmentation	Observed data likelihood	Eases explicit conditioning, simplifies likelihood computations, and enables extensions such as species misidentification
*Estimation procedure*	Reversible Jump MCMC(RJMCMC)	Fixed dimension Bayesian inference (e.g. using occupancy-like setup [22])	Straightforward implementation for non-uniform spatial support (e.g. unequal transect lengths)

Choices, alternative(s), and advantages of the modeling choices we made when analyzing double observer line transect data.

Using the true functional form for detection and habitat models, we sampled the posterior distribution with two Markov chains of length 270,000 with random starting values, recording posterior values from one out of every 20 iterations to save disk space. Convergence was determined by examining trace plots and other standard convergence diagnostics [32], and occurred after 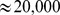 iterations. Removing the first 20,000 iterations as a burn-in and combining thinned versions of each chain resulted in 25,000 samples with which to conduct posterior inference.

#### Golf Tee Data

To further test our estimation approach, we analyzed data from an experimental survey of golf tees collected at the University of St. Andrews in 1999 [35]. This experiment mimics many nuances of actual animal transect surveys, but has the added advantage that true abundance is known. Several authors have previously used these data to gauge the performance of double observer transect estimators when estimating abundance from a real world dataset [7], [35].

The locations of 108 groups of green golf tees and 142 groups of yellow golf tees were randomly assigned over a landscape with two spatial strata (Fig. 3). Experiment designers placed 44 groups of green and 86 groups of yellow tees in the northern stratum (area 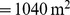), with the remainder being placed in the southern stratum (area 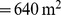). Tees were distributed in groups of 1–8 tees according to a predefined distribution (Fig. 4), and were further classified by level of exposure to surrounding grass, with some tee groups partially hidden by grass (Exposure = 0) and others more visible (Exposure = 1).

A total of 11 8-m wide transects were used to sample the population of golf tees, with eight independent observers traversing each transect. Transects varied in length, but completely covered the study area. We attempted to model these data in a similar manner to Laake and Borchers [7] (hereafter, LB), in order to make valid comparisons. Like LB, we pooled observations from observers 1–4 and 5–8 into two separate observation “teams” in order to investigate the performance of double observer methods in reconstructing true population size.

Using the double observer distance data, we attempted to estimate abundance of each “species” of tee (here, green and yellow) using our hierarchical probit formulation. We specified separate models for abundance intensity (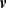) for each species, making each a function of stratum. For the probit of detection probability, we used the same model structure as selected by LB as having the most support in the data. Symbolically, this model expresses the expected value of detection probability (on the probit scale) as


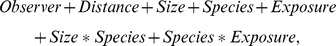


where *Size* specifies group size, and the ‘*’ symbol denotes a multiplicative interaction between variables. We modeled group size as a realization of an overdispersed, zero truncated Poisson process, and exposure as a categorical distribution.

We sampled the posterior distribution corresponding to the golf tee data with two Markov chains with different starting values. After an initial pilot run of 1000 iterations to adjust MCMC tuning parameters to desired ranges, each chain was run for 100,000 iterations. Inspection of trace plots and other standard MCMC diagnostics suggested that convergence to a stationary distribution was obtained almost immediately; as such, we combined the final 90,000 iterations of each chain together for inference.

## Results

### Simulation Results

Estimated abundances mirrored truth in each transect (Fig. 2). Estimated 95% credible intervals often included true parameter values (i.e. the values used to simulate data), although there were a few exceptions (Fig. 5). In particular, (i) the slope of the covariate-abundance relationship was overestimated for species one (posterior mean  = 1.10 instead of 1.00), and (ii) the magnitude of the distance effect on the detection function was consistently underestimated. We do not regard these discrepancies as overly problematic, however. Regarding (i), we were still able to capture the general pattern of linear increase in abundance (Fig. 2). Regarding (ii), we note that plots of realized detection functions still compare favorably to those used to generate data (Fig. 6).

### Golf Tee Results

For comparison with LB, we focus inference on the number of groups of animals (noting that posterior distributions for absolute abundance are also readily available). The posterior distribution for abundance of golf tees had a mean of 226 groups and 95% credible interval of (204, 251). By contrast, LB produced an estimate of 252, which was much closer to the true population size of 250. However, as LB note, this is somewhat accidental, as estimates of the number of groups in each color and exposure class differed substantially from true values. The hierarchical approach does better in this context, producing estimates that are as good or better than those generated by LB (Fig. 7). However, both approaches underestimate abundance for unexposed tees. We suspect that some of the unexposed tee clusters had very low (perhaps even zero) detection probabilities, and that the simple binary exposure covariate was insufficient to capture this variation.

Likelihoods for conventional mark-recapture distance sampling (MRDS) estimators are often written as a function of several different types of detection functions [7]. For instance, the *conditional* detection function, 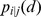 gives the probability that an object at distance 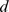 is detected by observer 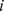 given that is was detected by observer 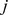. Similarly, the *individual* detection function, 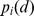 gives the unconditional probability that an object at distance 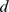 is detected by observer 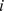; the *duplicate* detection function, 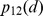 gives the probability that the object is detected by both observers; and the *pooled* detection function, 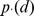 gives the probability that the object is detected by at least one observer. If observers are truly independent, there are theoretical relationships between these detection functions that should hold (e.g. 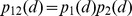); as such, examining plots of these detection functions can be informative when examining observer dependence. Similarly, Buckland et al. [9] defined a dependence parameter, 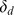 to express observer dependence as a function of distance, where 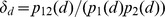. Although we have not included 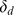 or any of these detection functions explicitly in our modeling efforts, we calculated these quantities post hoc using posterior means to help in interpreting our results. Observer dependence (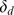) was estimated to be near 1.0 (that is, no dependence) for a large number of combinations of detection covariate values. Dependence was maximized with small group sizes, the second observer group, and green, unexposed tees (Fig. 8). This dynamic suggests heterogeneity in detection for small groups of unexposed tees that are far from observers.

As suggested by Royle [31], we were able to estimate a posterior mass function for group sizes in the population (Fig. 4), which compared reasonably with the known empirical distribution. Similarly, we were able to estimate the proportion of unexposed tees in the population; the posterior mean for green tees was 0.46 (95% CI: 0.30,0.61), and the posterior mean for yellow tees was 0.50 (95% CI: 0.40,0.60). By contrast, true values for this proportion were 0.56 and 0.53, respectively. Finally, we were able to estimate the effect of habitat covariates (in this case, strata) on abundance intensity.

## Discussion

Double-observer transect data are widely used to estimate abundance of animal populations. Although previously available estimators (notably, Horvitz-Thompson-like estimators [HT]; [35]) have proven remarkably versatile for estimating abundance from such data, they are of limited utility in making inference about the effects of ecological covariates on abundance, estimating the distribution of individual covariates in a population, and in making predictions about abundance in unsampled areas. The latter is especially important when it is not feasible to employ design-based statistical inference [1], either due to logistical or political constraints (e.g. certain areas are impossible to sample) or because the relative density of animals changes during the study [36].

An alternative approach to extrapolating abundance over a large spatial domain is to use a multi-stage statistical procedure, where the outputs from the first stage of modeling (e.g. density estimates) are used as inputs (data) for a second round of modeling (e.g. using a spatial model with habitat covariates) [37], [38]. This approach is widely used for extrapolating line transect estimates to unsampled areas in absence of a truly experimental sample design, particularly for cetaceans [39], [40]. However, care must be taken that variability associated with model outputs in the first stage of modeling are carried through when producing final abundance estimates [36].

We have presented a general framework for hierarchical analysis of double observer transect data that avoids many of these difficulties, obtaining posterior distributions for all parameters from a single analysis. In particular, abundance intensity can be made a function of habitat covariates, so that extrapolation to unsampled areas is straightforward (assuming that covariates are known for these areas). Further, precision of abundance estimates should be better than HT estimators whenever an explanatory habitat covariate can be identified. Modeling data from multiple observers allows us to relax the assumption of 100% detection on the transect line. Observer dependence can be accommodated via a bivariate normal distribution on the probit scale, helping to account for an increase in detection heterogeneity as a function of distance. To our knowledge, this is the first attempt at constructing a hierarchical model for double observer transect data.

Our model performed well in estimating abundance of two simulated populations whose abundance intensities were linearly and quadratically related to a hypothesized habitat covariate. Admittedly, we supplied the estimation model with the correct functional form for habitat relationships, a convenience typically not possible in real world estimation scenarios. Unfortunately, we know of no universally accepted method for conducting model selection among alternate functional forms for habitat-density relationships when using a RJMCMC approach to estimation. For instance, the popular deviance information criterion (DIC; [41]) has been shown to perform poorly in missing data applications [42]. Presently, we suggest using a flexible functional form, with a selection of habitat covariates guided by biology to parameterize these relationships. Analysts can then examine credible intervals to confirm whether such parameters are biologically meaningful. Alternative model selection procedures (e.g. based on posterior predictive loss criteria; [43]) are a subject of current research.

Our model also performed well when estimating the abundance of a known population of golf tees, in some cases outperforming conventional MRDS HT estimators. The estimates from both approaches (hierarchical, MRDS) tended to underestimate the number of golf tees that were visually obstructed; however, we suspect that this was largely due to some groups of tees being virtually undetectable. As such, this should not be seen as a failure of our proposed method, but as an artifact of the particular dataset. It is well known that transect data alone will produce negatively biased estimates if some subset of the population is unavailable for detection [44]; further elaborations are needed to arrive at unbiased estimates in these cases [45]–[47].

We made a number of modeling choices that differ from the way in which line transect data are typically analyzed. Some of these choices, together with our rationale, are listed in Table 2. One choice that deserves further explanation is our preference of the RJMCMC formulation for conducting inference in favor of an alternative “occupancy” parameterization recently advocated by several authors (e.g. [15], [16]). In a single site (or single transect) analysis, the two parameterizations are effectively identical, with the latter being more computationally efficient [16]. However, with multiple sites (or transects), the parameterization chosen for data augmentation can have practical ramifications for inference. A limiting case in spatial models is that counts (e.g. abundances in given transects) are distributed according to a Poisson distribution. The Poisson distribution has the convenient property of proportional scaling. For example, if transects differ in spatial support (i.e. are of different area), then abundance in a given transect 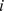 can be assumed to follow a 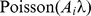 distribution. As we have shown in this paper, this relationship can be incorporated with relative ease using an RJMCMC data augmentation strategy (overdispersion relative to the Poisson distribution can be readily accommodated). By contrast, the approach advocated by Royle and Dorazio [15] and Link and Barker [16] requires use of an occupancy parameter, 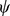, the interpretation of which requires reference to the value of 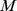 assumed in the model (e.g. 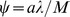). Further, the variance of expected abundance in this model (e.g. 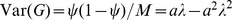) is negatively biased with regard to the Poisson variance (i.e. 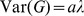). What effect this has on conducting hierarchical inference on abundance is presently unknown, but we recommend using the RJMCMC approach whenever spatial support differs among the areas sampled (e.g. when transects vary in size).

Although we are convinced that our approach is valuable for making predictions in unsampled areas, there is clearly need for more research in this area. By virtue of its hierarchical structure, our approach can easily be extended to incorporate spatial autocorrelation in abundance. For instance, Schmidt et al. [20] recently used conditionally autoregressive (CAR) models to account for spatial dependence in single observer transect data. However, predicting abundance with such models can be tricky, with spurious “edge effects” that can potentially compromise estimates of landscape-wide abundance [48]. Best practices for conducting posterior predictions with such models is a subject of current research.

We are also interested in extending our hierarchical framework to model partial observation and misclassification of species. In multi-species transect surveys, this is a real issue, as multiple observers often record species as unknown or have conflicting records. Currently available estimation approaches are incapable of handling such conflicts. Our data augmentation framework is clearly capable of treating true species as a latent (unobserved) variable, with misclassification introduced in the observation component of the model; however, parameter identification under such a scenario deserves further investigation.

We strongly encourage ecologists interested in abundance and species-habitat relationships to consider hierarchical modeling for estimation, especially when it is infeasible to conduct standard designed-based inference. When surveys are replicated across time and space, hierarchical models provide demonstrable advantages over design-based modeling approaches, as information can be shared across temporal and spatial domains [18], [22]. For multiple-observer transect surveys, our modeling framework and accompanying R package provides the tools necessary to implement a diverse array of hierarchical models.

## Supporting Information

Text S1 **Posterior sampling algorithm for multiple observer transect analysis.**
(PDF)
