# Supplementary material for: A Hierarchical Modeling Framework for Multiple Observer Transect Surveys
Source: PLoS One. 2012 Aug 8;7(8):e42294. doi: 10.1371/journal.pone.0042294 (PMC3414540; doi:10.1371/journal.pone.0042294)
Supplement: Text S1 — Posterior sampling algorithm for multiple observer transect analysis. (PDF) [file pone.0042294.s001.pdf]

Text S1: Posterior sampling algorithm for multiple observer transect analysis

Paul B. Conn [1,*], Jeffrey L. Laake [1], Devin S. Johnson[1],

1 National Marine Mammal Laboratory, Alaska Fisheries Science Center, National Marine Fisheries Service, Seattle, WA U.S.A.

∗ E-mail: paul.conn@noaa.gov

Here, we describe full conditional distributions and Markov chain Monte Carlo updating strategies for parameters of the hierarchical model for multiple-observer transect data.

Sampling $\boldsymbol{\beta}^{\text{hab}}$

The full conditional distribution for $[\boldsymbol{\beta}^{\text{hab}}|\ldots]$ (the conditional distribution of $\boldsymbol{\beta}^{\text{hab}}$ given all other parameters) is given by

$$[\boldsymbol{\nu}|\boldsymbol{\beta}^{\text{hab}}, \mathbf{X}^{\text{hab}}, \tau_\nu][\boldsymbol{\beta}^{\text{hab}}] \propto [\boldsymbol{\nu}|\boldsymbol{\beta}^{\text{hab}}, \mathbf{X}^{hab}, \tau_\nu],$$

where $[\boldsymbol{\nu}|\boldsymbol{\beta}^{\text{hab}}, \mathbf{X}^{\text{hab}}, \tau_\nu]$ is the product normal likelihood

$$\text{Normal}(\boldsymbol{\nu}; \mathbf{X}^{\text{hab}}\boldsymbol{\beta}^{\text{hab}} + \tau_\nu^{-1}\mathbf{I}). \quad (1)$$

Rewriting Eq. 1 as

$$\text{Normal}(\boldsymbol{\nu}; \mathbf{X}^{\text{hab}}\boldsymbol{\beta}^{\text{hab}}, \tau_\nu^{-1}\mathbf{I})$$

makes it into a standard linear regression formula. We may thus use Gibbs sampling to sample directly from $[\boldsymbol{\beta}^{\text{hab}}|\ldots]$ (e.g. [1], chapter 14), using

$$[\boldsymbol{\beta}^{\text{hab}}|\ldots] = \text{Normal}((\mathbf{X}'\mathbf{X})^{-1}\mathbf{X}'(\boldsymbol{\nu}-), \tau_\nu^{-1}(\mathbf{X}'\mathbf{X})^{-1}) \quad (2)$$

(temporarily replacing $\mathbf{X}^{\text{hab}}$ with $\mathbf{X}$).

Sampling $\boldsymbol{\nu}$

We used different strategies for updating ν_s depending on whether or not sampling was conducted in strata s. To update ν_s for cells where sampling was not conducted, we simulated ν_s directly using

$$\nu_s \sim \text{Normal}([\mathbf{X}^{\text{hab}}\boldsymbol{\beta}^{\text{hab}}]_s, \tau_\nu^{-1}).$$

We iteratively updated the remaining elements of $\boldsymbol{\nu}$ using Metropolis-Hastings step, with a target acceptance rate of 30-40%. The full conditional distribution for ν_s needed to perform Metropolis-Hastings updates for sampled cells is given by

$$[\nu_s|\ldots] = \text{Normal}\left(\nu_s; [\mathbf{X}^{\text{hab}}\boldsymbol{\beta}^{\text{hab}}]_s, \tau_\nu^{-1}\right) \text{Poisson}\left(G_s; \exp(\nu_s)\right).$$

Sampling τ_ν

We directly sample τ_ν may using

$$\tau_\nu \sim \text{Gamma}\left(0.5S + \alpha_\nu, 0.5\boldsymbol{\mu}_\nu'\boldsymbol{\mu}_\nu + \beta_\nu\right),$$

where $\boldsymbol{\mu}_\nu = \mathbf{X}^{\text{hab}}\boldsymbol{\beta}^{\text{hab}}$.

Sampling $\boldsymbol{\beta}^{\text{det}}$

Using Eq. 2 to transform bivariate responses into univariate responses when necessary, i.e.,

$$\acute{Y}_{ijk} = \begin{cases} \tilde{Y}_{ijk} & O_j = 1 \\ \tilde{Y}_{ijk} - \rho_{ij}\tilde{Y}_{ij,3-k} & O_j = 2 \end{cases},$$

we can show that

$$\acute{\mathbf{Y}} \sim \text{Normal}(\mathbf{X}\boldsymbol{\beta}^{\text{det}}, \boldsymbol{\Sigma}),$$

where $\mathbf{X} = \mathbf{X}^{\text{det}}$ if $O_j = 1$ and

$$\mathbf{X} = \begin{bmatrix} \mathbf{X}_1^{\text{det}} - \rho\mathbf{X}_2^{\text{det}} \\ \mathbf{X}_2^{\text{det}} - \rho\mathbf{X}_1^{\text{det}} \end{bmatrix}$$

if $O_j = 2$ (Here, $\mathbf{X}_k^{\text{det}}$ designates the design matrix for the kth observer). Also, $\boldsymbol{\Sigma}$ is a diagonal matrix with entries corresponding to $1-\rho_{ij}^2$ for each response variable. Under this formulation, we can use basic Gibbs sampling for regression with a known covariance matrix (e.g., [1], Eqs. 14.11-14.12) to sample from $[\boldsymbol{\beta}^{\text{det}}|\ldots]$. In particular,

$$[\boldsymbol{\beta}^{\text{det}}|\ldots] = \text{Normal}\left((\mathbf{X}'\boldsymbol{\Sigma}^{-1}\mathbf{X})^{-1}\mathbf{X}'\boldsymbol{\Sigma}^{-1}\acute{\mathbf{Y}}, (\mathbf{X}'\boldsymbol{\Sigma}^{-1}\mathbf{X})^{-1}\right).$$

Sampling ρ

Letting ξ denote the set of transects for which $O_j = 2$ (i.e., the set for which there were two observers present), we used a Metropolis-Hastings step to update ρ. The full conditional distribution for this update is given by

$$[\rho|\ldots] \propto \left[\prod_{j\in\xi}\prod_{i=1}^{G_{ij}}\{(1-\rho_{ij}^2)\exp\left((1-\rho_{ij}^2)^{-1}(\Delta_{ij1}^2+\Delta_{ij2}^2-2\rho_{ij}\Delta_{ij1}\Delta_{ij2})\right)\}\right]^{-0.5},$$

where $\Delta_{ijk} = \tilde{Y}_{ijk} - [\mathbf{X}^{\text{det}}\boldsymbol{\beta}]_{ijk}$.

Sampling $\tilde{\mathbf{Y}}$

For animals currently in the population (i.e. for $i \in [1, 2, \ldots, G_j]$), the full conditional distribution $[\tilde{Y}_{ijk}|\ldots]$ is a truncated normal, and may be sampled directly. If $Y_{ijk} = 0$, $\tilde{Y}_{ijk}$ is drawn from a normal distribution with the constraint that $\tilde{Y}_{ijk} < 0$, while $\tilde{Y}_{ijk} > 0$ if $Y_{ijk} = 1$. The corresponding untruncated distribution is given by

$$\tilde{Y}_{ijk} \sim \text{Normal}\left([\mathbf{X}^{\text{det}}\boldsymbol{\beta}^{\text{det}}]_{ijk}, 1\right)$$

when $O_j = 1$, and

$$\tilde{Y}_{ijk} \sim \text{Normal}\left([\mathbf{X}^{\text{det}}\boldsymbol{\beta}^{\text{det}}]_{ijk} + \rho_{ij}(\tilde{Y}_{ij,3-k} - [\mathbf{X}^{\text{det}}\boldsymbol{\beta}^{\text{det}}]_{ij,3-k}), 1-\rho_{ij}^2\right)$$

when $O_j = 2$.

Sampling $\boldsymbol{\theta}, \boldsymbol{\epsilon}$

If random effects are not specified (i.e. $\epsilon_{ijk} = 0 \; \forall \; \{i, j, k\}$), specification of conjugate priors for the Poisson, zero-truncated Poisson, and multinomial distributions meant that full conditionals were available in closed form and could be simulated directly. If random effects are specified, both the parameters of the underlying distribution (i.e. $\boldsymbol{\theta}$) and the random effects themselves (i.e. $\boldsymbol{\epsilon}$) are sampled via the Metropolis-Hastings algorithm. Owing to the relatively large number of individual covariate distributions, we refer the reader to Dataset S1 for further details.

References

1. Gelman A, Carlin JB, Stern HS, Rubin DB (2004) Bayesian Data Analysis, 2nd Edition. Boca Ration: Chapman and Hall.
